# Psychomotor Retardation in Depression: A Systematic Review of Diagnostic, Pathophysiologic, and Therapeutic Implications

**DOI:** 10.1155/2013/158746

**Published:** 2013-10-30

**Authors:** Djamila Bennabi, Pierre Vandel, Charalambos Papaxanthis, Thierry Pozzo, Emmanuel Haffen

**Affiliations:** ^1^Department of Clinical Psychiatry, University Hospital of Besançon, 25030 Besançon, France; ^2^EA 481 Neuroscience, IFR 133, University of Franche-Comte, 25030 Besançon, France; ^3^INSERM U1093 Cognition, Action, et Plasticité Sensorimotrice, University of Bourgogne, UFR STAPS, 21078 Dijon, France; ^4^University of Bourgogne, UFR STAPS, 21078 Dijon, France; ^5^Institut Universitaire de France, University of Bourgogne, 21078 Dijon, France; ^6^Clinical Investigation Centre CIC-IT 808 INSERM, University Hospital of Besançon, 25030 Besançon, France

## Abstract

Psychomotor retardation is a central feature of depression which includes motor and cognitive impairments. Effective management may be useful to improve the classification of depressive subtypes and treatment selection, as well as prediction of outcome in patients with depression. The aim of this paper was to review the current status of knowledge regarding psychomotor retardation in depression, in order to clarify its role in the diagnostic management of mood disorders. Retardation modifies all the actions of the individual, including motility, mental activity, and speech. Objective assessments can highlight the diagnostic importance of psychomotor retardation, especially in melancholic and bipolar depression. Psychomotor retardation is also related to depression severity and therapeutic change and could be considered a good criterion for the prediction of therapeutic effect. The neurobiological process underlying the inhibition of activity includes functional deficits in the prefrontal cortex and abnormalities in dopamine neurotransmission. Future investigations of psychomotor retardation should help improve the understanding of the pathophysiological mechanisms underlying mood disorders and contribute to improving their therapeutic management.

## 1. Introduction

Psychomotor retardation (PMR) has been recognized as one of the most fundamental features of major depressive disorder by the earliest psychiatric authors and is reflected in the use of various contemporary classification systems [1–3]. Descriptions of PMR have remained consistent in the literature; most portrayals of depressive symptomatology emphasised disturbances in speech, facial expression, fine motor behaviour, gross locomotor activity, or ideation [4–6]. Since the end of the 20th century, several authors have argued that the presence of clinical PMR allows determining clinically meaningful depressive subtypes (melancholic with and without psychotic features, bipolar and unipolar disorders) [7–9]. Other authors have proposed that motor retardation reflects a fundamental dimension of depression [4, 10]. Moreover, motor disturbance in depression may indicate an underlying neuropathology and could be relevant in the context of therapeutic interventions [5]. Although psychomotor disturbances are included in most diagnostic systems and probably have prognostic and pathophysiological significance, explicit definitions of psychomotor phenomena remain elusive [5, 11]. In order to specify the significance of psychomotor symptoms across the full spectrum of depressive disorders, experimental methods investigating motor and cognitive components of PMR have been developed. Objective psychomotor assessments may improve classification, longitudinal monitoring, treatment selection, and prediction of outcome in patients with depression.

The aim of this paper was to review the current status of knowledge about PMR in depression. 

Our review focuses on empirical studies seeking to objectively quantify the manifestations of PMR. In addition, we have provided an overview of some of the conceptual and empirical backgrounds related to the pathophysiologic significance and the predictive value of PMR. 

## 2. Method

Three electronic databases were searched to identify relevant manuscripts: PubMed/Medline, Cochrane, and PsycInfo. Our initial search strategy included one main term, namely, “depressive disorder,” combined with the following: ≪psychomotor retardation≫, ≪motor activity≫, ≪psychomotor disorders≫, and ≪perceptual motor processes≫. The reference lists of the selected manuscripts were scrutinised for additional studies.

Studies were limited to human studies reported in English and were eligible for inclusion if they addressed both depression and retardation symptoms. Articles were included if they contained primary data derived from clinical trials or longitudinal or cross-sectional studies. Excluded studies were those addressing depression due to specific disease processes (e.g., Parkinson's disease or dementia). We initially applied the above eligibility criteria to the citations and abstracts generated by the search. Based on this information, we excluded publications not meeting the inclusion criteria. When an article met the inclusion criteria, or when there was not sufficient information to definitely exclude it, we retrieved the full text. We then reviewed these potentially relevant articles to determine whether the inclusion criteria were in fact met. Of the 144 papers where full-text articles were reviewed, we excluded a total of 28 articles; 24 studies did not meet eligibility criteria, and 4 presented duplicate data. Thus, we obtained data from 116 papers that met our eligibility criteria.

The reviewed studies are listed in Tables 1, 2 and 3, according to sample, design, measure, and results. Diagnoses were more often based on DSM or Research Diagnostic Criteria. The main observations are that (i) most samples sizes are relatively large, (ii) the majority of the studies include a control group, (iii) the assessment methods and outcomes measures differed substantially across studies, and (iv) there were few discrepancies in the findings, mainly due to the homogeneity of the methodology.

## 3. Results

### 3.1. Assessments of PMR

Longitudinal investigations of motor behaviour are complicated by the effects of confounds such as motivational factors, psychotropic medication, or time of assessment [12]. Motivational factors including interest, pleasure, and reactivity to pleasurable stimuli contribute to the initiation and progression of motor activity and may interfere with the expression of retardation [13, 14]. Circadian rhythms are another confounding factor, with retardation being more pronounced during the morning than in the evening. Psychoactive medication may have disruptive effects on psychomotor functioning, causing sedation or impairment in psychomotor and cognitive function [15].

#### 3.1.1. Clinical Assessments

Clinical rating scales of depression typically include only one item for psychomotor disturbance, and cognitive or motor aspects of agitation and retardation are intermixed [16–18]. The Hamilton Depression Rating Scale (HDRS), for example, contains only two of the 17 items assessing psychomotor symptoms. Retardation also appears indirectly in several different items concerning fatigue, loss of energy, or lack of concentration. Three scales have been specifically designed to assess PMR in depression, each addressing different objectives: the Salpetrière Retardation Rating Scale (SRRS), the CORE index of melancholia, and the Motor Agitation and Retardation Scale (MARS) [19–21].

*(a) The Salpetrière Retardation Rating Scale (SRRS)*. The SRRS, developed by Widlöcher, focuses on motor and cognitive aspects of retardation. This author considered PMR as a unique global process including motor and psychic observational disturbances. The SRRS has been used in a number of studies to measure severity of psychomotor retardation or its capacity to predict response to antidepressant medication. This scale contains fifteen items, each scored from 0 (normal) to 4 (severe) with a total score range of 0 to 60. The first 6 items gauge different manifestations of motor retardation such as slowness of gait, limb movement, or speech. The next two items are designed to objectively assess cognitive function, whereas the last 5 are related to subjective appreciation of cognitive activities. One additional item proposed a global evaluation of psychomotor retardation. Factor analysis suggested a three-factor solution: the first factor, including all items, accounted for 60% of the cumulative variance; the second factor was composed of SRRS items 1 to 5, which are motor retardation items; the third factor was composed of items which grade subjective experience of retardation and is influenced by anxiety. Correlations between SRRS and HDRS (*r* = 0.58) or MADRS (*r* = 0.68) have indicated good convergent validity [19]. Otherwise, the SRRS has been found to have strong correlations with motor and cognitive measures of retardation such as speech pause time [22, 23], level of activity [10, 24–26], or reaction time. Bonin-Guillaume et al. designed the Retardation Rating Scale (RRS) to evaluate the global aspect of PMR in the geriatric population [27]. The RRS includes items related to motor and cognitive retardation and differs from the SRRS with one additional item rating motility and one less rating speech. This scale has proven to have good psychometric properties in subjects who are over 80.

*(b) The CORE Index*. This measure was designed to sybtype depressed patients into melancholic and nonmelancholic groups and supports the hypothesis that retardation and agitation symptoms distinguish endogenous psychotic depression from neurotic and reactive depression. [7, 21]. As instrument rates are used to assess observed behavioural nuances, clinical experience with depressed patients (especially the severely depressed) is required. The CORE index is composed of 18 items, scored on a 4-point scale. A score of 0 indicates that the sign is absent or trivial, while scores of 1 to 3 indicate definite presence with increasing severity. The total score range of 0 to 54 and a cut-off score of 21 are used to classify melancholic depression. Factor analysis showed three interpretable domains: (1) retardation items (52% of variance), (2) agitation items (15% of variance), and (3) noninteractiveness (5% of variance). The factor ≪noninteractiveness≫ refers to items, ≪length of verbal responses≫ and ≪poverty of associations≫. Further CORE scores are associated with nonsuppression of cortisol following overnight dexamethasone [85] electronic measures of neuropsychological slowing [119]. This scale has a good structural validity, good internal consistency, and convergent validity. The CORE index is used to quantify the degree of psychomotor impairment or to constitute subgroups of patients suffering from melancholic depression.

*(c) The Motor Agitation and Retardation Scale*. MARS was developed to provide a measure of 19 abnormal behaviours associated with agitation and retardation in depressive disorders [20]. This scale included observable motor signs in five domains: trunk, limbs, eyes, face, and voice. Each item is scored from 0 to 4 depending on the presence or severity of symptoms. The MARS offers a rapid clinical assessment of motor signs but does not include items concerning noninteractiveness and cognitive slowing. 

Although these three scales were developed to measure similar constructs, they have a number of differences. The SRRS attempts to directly tap into the mental life of the subject, specifically the presence or absence of perceived mental slowing. By contrast, the CORE scale does not rely on verbal responses from the patient, allowing it to be scored even in stuporous or mute patients [109]. 

#### 3.1.2. Objective Measures

*(a) Speech*. Alterations in paralinguistic aspects of speech, associating changes in fluency and prosody, are a traditional finding in depressed patients. Earlier investigators simply used speech samples extracted from audiotape recordings of semistructured interviews or counting tasks [22, 23, 28, 29, 32]. Szabadi et al. found a significant elongation of speech pause time in their depressed patients, whereas the phonation time remained constant. Other groups replicated this finding in larger samples of unipolar or bipolar depressed patients. More recent studies, using a sophisticated computer-driven acoustic measuring system, confirmed reduced prosody by analysing the variables measuring aspects of fundamental frequency [30, 31, 34, 35]. Moreover, differences between depressed and normal comparison groups have also been shown in articulation characteristics [33]. Several studies have found a strong correlation between change in clinical ratings of symptom severity and several key voice acoustic measures. These measures appear to be sensitive to both early symptomatic improvement and degree of response to therapeutic intervention and may be state-dependent indicators of depression [23, 34].

*(b) Gross Motor Activity*. Psychomotor abnormalities are manifest in various motor domains including alterations of gait, posture, or limb movement and have been investigated in patients by actigraphy, cinematography, or ground reaction forces [3]. Ratings of nonverbal behaviour in depressive states showed altered temporal segmentation of movements, increased brief repetitive body touching, and continuous hand-to-head and hand-to-hand touching [42]. Recordings of reaction times and velocity scaling during wrist flexion, using a hand-held rotation sensor, have been used to explore the subject's ability to increase movement velocity [98]. Actimetry is another relatively simple method of spontaneous motor activity exploration. Applied to depression it allows a quantitative and qualitative study of patterns of activity through different parameters distinguishing periods of rest and activity. Studies included in our review offer an analysis of the average level of activity and parameters of immobility for different periods [25]. Several authors have reported variations on the circadian expression of psychomotor symptoms in depression, and observed a general disorganization of motor activity, with a significant reduction in activity levels in the late morning, early afternoon, and early evening [13, 24–26, 97]. In depressed patients, lower motor activity levels were associated with clinical ratings of retardation (*r* = 0.717, *P* < 0.05) and depression severity (*r* = 0.66, *P* < 0.05) [25]. The study of the patient's environment (hospital setting instead of home environment) has influenced the type of daily activities and consequently the 24 h pattern of motor activity. Global motor activity has also been objectively analysed during locomotion. Spatiotemporal gait parameters were measured during overground walking at self-selected walking speed on a walkway. Compared to controls, depressed patients showed significantly lower gait velocity, reduced stride length, double limb support and cycle duration [36–39]. There was a significant correlation found between cadence and gait velocity in depressed patients (*r* = 0.51, *P* < 0.05). These studies have failed to find a correlation between spatiotemporal gait parameters and clinical assessment of motor retardation or severity of depression.

*(c) Fine Motor Activity*. Sensitive instrumental measure of motor slowing may allow detection of motor system abnormalities that are not clinically observed.

*(1) Drawing Tasks*. Kinematic analysis of drawing and handwriting movements allows precise and objective studies of motor abnormalities in depression [44, 46–49]. These studies are based on computerised recording techniques (graphics tablet and a pressure-sensitive pen) to analyse motor parameters during the copying of simple or complex geometric figures. The instructions given to subjects included particular requirements for accuracy and speed of movement. Specific variables that allow a distinction between cognitive and motor processes included both initiation time and movement time. Motor slowing affects both the motor and cognitive processes, as reflected by an increase in motor and initiation time during simple and complex tasks [44, 46, 47]. Furthermore, the melancholic patients were more severely affected than the nonmelancholic patients [47]. Another approach, based on the investigation of internally and externally cued movements, aimed to delineate psychotic major depression in the fine motor domain. Hoffstaedter et al. performed a computerised motor paradigm and reported that internally cued movements were more severely affected than externally cued reactions during major depressive disorder (MDD), suggesting specific impairments of visuospatial and attentional processing as cognitive aspects of psychomotor functioning [50].

*(2) Eye Movements.* Eye movement tasks constitute an interesting tool to investigate psychomotor functioning, through exploration of basic and high levels of motor control including spatial working memory, prediction, and response suppression. A number of studies have explored eye movements in groups of patients with major depression. These studies have reported the presence of intact reflexive saccades [52, 53], normal latencies and velocities for voluntary saccades, but increased durations [52], normal or slightly increased rates of response suppression errors, and reduced accuracy for memory-guided saccades [52]. In addition, one study has explored differences in eye movements between melancholic and nonmelancholic patients and showed in the melancholic patient greater intrasubject variability of latencies, lower peak saccade velocities, and reduced accuracy of the primary saccade [54].

*(3) Facial Movements*. Recordings of facial muscle electromyographic activity during the generation of affective imagery represents one promising strategy for examining motor deficits in depression [5]. In contrast to controls, depressed subjects showed significant reduced electromyographic patterns for happiness [51]. Retardation was documentable even though trained clinicians were unable to identify obvious clinical signs of motor retardation.

*(d) Cognitive Measures*. Slow ideation is a common subjective complaint in patients with motor retardation [59]. Different studies have sought to determine to what extent certain neuropsychological tests might be more specifically related to retardation than to general cognitive inefficiency [55, 56, 58, 63]. 

Reaction time methods have been used as a simple and objective index of PMR. Numerous studies have also demonstrated the independence of central (cognitive) and peripheral (motor) components of reaction time by separately measuring the time required to initiate a response (decision time) and the time required to carry out the motor activity to complete the response. Cornell et al. found a motor component to PMR in both melancholic and nonmelancholic groups, while only melancholic patients manifested an additional cognitive impairment [56]. Using the Nufferno speed test and the Gibson maze test, Blackburn observed a significant reduction of psychomotor speed in their depressed bipolar patients [55]. Bonin-Guillaume et al. [63] performed two reaction time experiments using an additive factor analysis and found that PMR associated with depression was limited to the components of response selection and motor adjustment.

Measurements of ideational retardation, and notably latency of response to the Rorschach plates and to simplified nonfigurative line drawings, have been proposed by Brebion et al. [59]. These authors have shown significant correlations between the latency of response and scores on the SRRS (*r* = 0.71, *P* < 0.0005). Finally, a modified version of Posner's covert orientation of visual attention test, wich involves shifting of preparation for response from on side to another, was designed by Smith [58]. This author found that the time of maximal response preparation occurred later in depressive patients than in controls, and was strongly correlated with observable psychomotor retardation but not with depressive severity. Mental rotation, as a reflection of visuospatial cognitive operation, is another useful tool for assessing motor preparation. During ego-rotation and object-rotation tasks, involving the creation of a mental image of an object and its subsequent rotation, MDD patients exhibited specific deficits compared to controls [64].

### 3.2. Factors Influencing PMR

#### 3.2.1. Influence of Age

In later life, age and depression may interact, resulting in a more pronounced retardation in geriatric patients. PMR occurs in atypical depression presentations in the elderly, such as subsyndromal depression [120] or depression-executive dysfunction syndrome [121]. These motor abnormalities differed from slowing due to normal aging in that only some information-processing stages were affected by age whereas all the processing stages were affected by age [27]. Furthermore, experimental studies have demonstrated that depressed geriatric patients exhibited PMR similar to younger adults [34, 48, 122]. Considering the presence and type of psychomotor disturbance may be an important psychopathologic feature that differentiates clinically distinct forms of juvenile MDD. Moreover, Leventhal et al. identified agitated and agitated-retarded depression as a specific phenotypic syndrome in young adults [123].

#### 3.2.2. Influence of Sex

Studies of gender differences in the clinical presentation of depression have provided divergent results. While some authors found no clinical relevant gender differences in the prevalence of any psychomotor symptoms [124], other studies reported higher rates of retardation in males than females [125, 126]. The discrepant findings between studies may relate to differences in methodology, sample sizes, and severity or nature of the depressive disorder. 

#### 3.2.3. Influence of Treatments

Pharmacological treatments can contribute to improve psychomotor functioning, but may also have disruptive effects, causing sedation or impairment in psychomotor and cognitive function. Benzodiazepine may affect the speed with which simple repetitive motor actions are performed [127] and impaired performances during a tapping task [128]. Clinical practice suggests that motor slowing is quite frequently found in patients treated with classical neuroleptics, although no effects or even improvements have been found [129].

#### 3.2.4. Influence of Depression Subtype

*(a) Melancholic Depression*. In a series of articles devoted to the study of psychomotor disturbances during melancholic access, Parker et al. proposed PMR as a marker of an underlying neuropathological process specific for the melancholic depressive subtype [21, 119, 130]. The biological and clinical plausibility for this putative endophenotype include associations between psychomotor disturbances and hypothalamic-pituitary-adrenocortical axis dysfunction in depressed subjects, and parkinsonian movement deficits in melancholic patients [7, 85]. Unfortunately, information on heritability, familial association, state-independence, and cosegregation of specific psychomotor disturbances are lacking [131]. Comparing melancholic and nonmelancholic samples, several researchers found that melancholic patients are more retarded during drawing or oculomotor tasks [47, 54], limb movements [68], or reaction time [56]. However, other authors failed to reproduce these results [30, 132].

Concerning melancholic depression with a psychotic feature, Parker et al. suggested that, in addition to the presence of delusions and/or hallucinations, the presence of psychomotor disturbances was the most consistently reported additional feature. Psychotic depression appears to be most specifically associated with profound noninteractiveness and severe agitation.

*(b) Bipolar/Unipolar Depression.* Psychomotor retardation is considered a robust feature distinguishing between bipolar and unipolar depression, supporting historical clinical description considering psychomotor disturbance as a cardinal feature of bipolar depression [8]. Nevertheless, several studies found no differences in rates of retardation between major depressive disorder and type 2 bipolar disorder samples, but rather significantly higher rates of agitation among the group with type 2 bipolar disorder [133, 134]. PMR, diagnosed by clinical observation or experimental assessments, may have more relevance to bipolar type I depression.

### 3.3. Specificity of PMR in Depression

Psychomotor retardation can be present in different neuropsychiatric disorders, including schizophrenia or Parkinson's disease. For instance, experimental comparisons between depressive and schizophrenic patients have shown a different slowing structure, with a slowness in both motor and cognitive components in depressed patients, whereas the schizophrenic patients only exhibited a slowness in the cognitive component [66]. Similarly, patients with depression exhibit difficulties initiating movements in the absence of external cues, as patients with Parkinson's disease. Slowed motor activity and difficulties in self-initiating movements, common to melancholic depression, negative symptoms in schizophrenia, and Parkinson's disease, may reflect dysfunction within frontostriatal circuits [54]. 

Correlation analyses between motor signs and severity of depression indicate that some aspects of psychomotor slowing are related to state changes in depression [12] Szabadi et al. [28]. This notion is supported by positive effects of pharmacological treatments, leading to changes in depression rating scale scores significantly correlated with changes in motor performances Sabbe et al., Volkers et al. [92, 135]. However, other findings suggest that some components of motor retardation are trait characteristics in depression. For example, Caligiuri and Ellwanger [65] found specific abnormalities on a velocity scaling measure in bipolar depressed patients, compared with unipolar patients. 

To date, the data available provide arguments in favour of the two hypotheses, and this question still remains to be investigated.

Our review of the literature illustrates the multiplicity of objective exploration of PMR in different areas of psychomotor functioning. These studies support the achievement of cognitive and motor functions involved in the production process of the movement. Correlations between objective measures and subjective appreciation of PMR based on clinical scales have led to conflicting results. These results may be related to differences in clinical expression of retardation or even to the existence of a subclinical slowdown not identifiable by the clinician. There are no published studies to our knowledge combining several techniques of PMR objective measures. The combination of different experimental techniques for measuring retardation with clinical assessments could offer an increased understanding of PMR in depression. 

### 3.4. Neuropathology

The neurobiology of major depressive disorders has been associated with alterations in prefrontal and orbitofrontal cortices, anterior cingulate, amygdala, and the hippocampus [136, 137]. Concerning the neurobiology of motor retardation in depression, some findings point to structural alterations of the basal ganglia circuits [76]. Although the association between Parkinson's disease and bradyphrenia is uncertain, similarities have been drawn between the motor slowness of PMR in some major depressed patients and bradykinesia in Parkinson's disease and consequently to the possibility that the two phenomena may share some common underlying pathology [33, 65, 67]. The basal ganglia system constitutes, therefore, a possible candidate as a site of motor dysfunction common to these two disorders. Various experimental works have suggested that some aspects of motor deficits are equally present in the two pathologies. In one study two groups of patients exhibited similar deficits in self-initiated movements [67], in the programming of movement velocity [65, 68], or in articulation [33]. Moreover, patients with MDD and PMR were shown to have reduced extracellular dopamine in caudate and putamen. Martinot et al. assessed presynaptic dopamine function by using positron emission tomography (PET) and 6-[18F] fluorodopa in depressed patients and healthy subjects. [18F] DOPA uptake Ki values in the left caudate were significantly lower in patients with psychomotor retardation than in comparison subjects [82]. Meyer et al. obtained concordant results, and observed an elevation in D2 binding in caudate and putamen, measured with [11C] raclopride PET [83]. Shah et al. reported decreased dopamine function, as indexed by increased binding of the dopamine D 2/3 ligand 1-123-IBZM, in the right striatum if patients with major depression [80]. Conversely, one study yielded no evidence to support the hypothesis that patients with psychomotor retardation have decreased dopaminergic function [81]. Beside dopaminergic dysregulation, some studies have linked psychomotor symptoms and noradrenergic transmission [138]. Evidence of clinical activity of antidepressant drugs with noradrenergic action supported this hypothesis. The interaction of GABAergic system and psychomotor retardation was supported by Bajbouj et al. who found a strong correlation between psychomotor retardation measured with the CORE questionnaire and transcranial magnetic stimulation (TMS) based measures of cortical excitability [84].

Neuroimaging studies provide strong evidence for an involvement of brain structures associated with initiation, planification, and motor control of behaviour in clinical psychomotor symptoms. Negative correlations between cerebral blood flow and clinically rated PMR were found in the dorsolateral prefrontal cortex, orbitofrontal cortex, and angular gyrus [72, 75, 76]. Similarly, longer reaction times were associated with reduced cerebral blood flow (CBF) increase in the striatum during a simple motor task [74]. In addition, structural imaging studies have indicated a negative correlation between white matter hyper intensities and psychomotor speed [73]. Walther et al. linked PMR to CBF of the supplemental motor area, suggesting disbalanced motor control in MDD [77, 78]. More recently, studies using diffusion tensor imaging measures of white matter pathways connecting these regions demonstrated altered white matter organisation of rostral anterior cingulate cortex-pre-supplementary motor area and dLPFC-presupplementary motor area pathways [78].

### 3.5. Treatments

Several studies suggest that PMR measures may provide prognostic information concerning antidepressant response. The results of clinical rating suggest that PMR can be used as marker for antidepressant response. Flament et al. found that their patients with motor retardation responded less favourably to 6 weeks of fluoxetine or sertraline treatment compared to nonmotor retarded sample of patients [93]. Sechter et al. found that baseline retardation predicted a response to milnacipran. Similarly designed studies found that PMR failed to predict a response to selective serotonin reuptake (SRRI) [90, 99]. Experimental assessments of PMR support his predictive value. In an open-treatment medication study, Caligiuri et al. demonstrated that a quantitative measure of motor programming may be a useful predictor of antidepressant nonresponse [98]. Concerning information processing speed, Dunkin et al. found that patients whose symptoms did not remit following 8 weeks of fluoxetine treatment had impaired baseline pretreatment functioning [139]. Conversely, Taylor et al. suggest a deficit in psychomotor speed distinguishing SSRI nonresponse [100].

The impact of different pharmacological treatments on motor symptoms in depressed patients was examined using clinical scales or experimental assessment. Ferguson et al. combined the data of 4 clinical studies and found a significant improvement of PMR in patients following 4 weeks of reboxetine treatment [96]. In their meta-analysis, Entsuah et al. reported beneficial effects of venlafaxine [91]. The preferential efficacy of venlafaxine for psychomotor retardation symptoms was recently supported by Singh et al. in a study comparing the clinical effects of venlafaxine and escitalopram in MDD [103]. Comparing clinical response, Del Zompo et al. observed that minaprine was more effective than tricyclic antidepressant on PMR in depressed patients [88]. Assessing the therapeutic efficacy of various antidepressants (minaprine, amineptines and clomipramine) Rampello et al. obtained convergent results [89]. In a double blind study, Bondareff et al. observed similar response rate to sertraline and nortriptyline while Navarro et al. found a better response rate for nortriptyline than citalopram in an elderly depressed patient [94, 95]. In addition, Sabbe et al. found that the effect of fluoxetine on the motor components of drawing were relatively low [92]. Unfortunately we failed to find available data on effects of psychotherapy on PMR.

The latency of action of antidepressive medications or their contraindication justifies the use of electroconvulsive therapy (ECT) in first intention in severe depressive states where the prognosis is committed. Joining the notion of severity, depressions with delusions and those with catatonic symptoms are preferential indications of ECT, as well as melancholic depression, due to gravity or clinical criteria. The analysis of the association of specific symptom profiles with ECT outcome indicates that the psychotic feature, older age, and psychomotor disturbances were predictive of greater response [108, 109, 112]. 

A combined analysis of randomized controlled trials of ECT versus simulated ECT showed that real ECT had a therapeutic advantage, specifically among patients with retardation and/or delusions [110]. Nevertheless, in 2 randomized controlled trials, involving 148 patients, Sobin et al. investigated the utility of depression subtypes in predicting ECT response and concluded that ECT was a treatment option for patients with major depression; however, neither the presence of psychotic features, retardation, and/or agitation predicted superior response [111].

More responsible for the variation of results among studies on ECT can be, respectively, the number of ECT sessions applied, the methodological weakness of some studies that did not specify the electrical parameters of the bilateral ECT and/or unilateral ECT.

Only four studies have investigated whether HF-rTMS treatments affect psychomotor symptoms [113–115]. Baeken et al. did not report any significant relationship between psychomotor symptoms and clinical response. Moreover they observed a reduction of psychomotor disturbances after the treatment, independent of age, sex, and duration of illness [115]. Hoppner et al. obtained convergent results, with a reduction of the score on the MARS scale after treatment [113]. In a sample of severely depressed patients, Ullrich et al. reported a significant improvement of processing speed performance, which covaried with the improvement of psychomotor retardation, after 3 weeks of ultrahigh frequency rTMS [116].

Concerning behavioural facilitatory effects of transcranial direct current stimulation (tDCS), Loo et al. failed to predict the antidepressant response using the CORE measure in two subsequent studies. However, they obtained positive results on depressive symptomatology, with a greater reduction in MADRS scores after real versus sham stimulations after applying 15 sessions of anodal tDCS at 2 mA to 64 unipolar and bipolar depressed patients [117, 118].

## 4. Conclusion

Psychomotor retardation is a central feature of depression that can have clinical and therapeutic implications. This includes both motor and cognitive impairments, affecting speech, motility, and ideation. These symptoms may severely impact patient's psychosocial functioning [140, 141] and are closely linked with severity of depression [9]. 

The still fragmentary data on the status of PMR in depression confirm the need for further quantitative and qualitative investigations, particularly concerning its relationship with motivation and emotions. 

The study of the dynamic interactions of systems governing motor, cognitive, and emotional aspects of movement production is likely to enrich the understanding of the neurobiological substrates of depression and its treatment. 

## Figures and Tables

**Table 1:** Studies exploring experimental assessments of psychomotor retardation.

Authors	Sample (criteria)	Treatments	Measure	Variables
Szabadi et al. 1976 [28]	Depressed = 4 (NS) Controls = 4	Amitriptyline	Automatic speech HDRS	Speech pause time Phonation time
Greden et al. 1981 [29]	MDD = 36 (RDC) 24 UP/12 BP Controls = 19	NS	Automatic speech HDRS	Speech pause time Phonation time
Hardy et al. 1984 [22]	MDD = 16 (DSM III)	Clomipramine Mianserin ECT	Automatic speech HDRS SRRS	Speech pause time Phonation time
Hoffmann et al. 1985 [23]	MDD = 22 (RDC) 12 UP/10 BP Controls = 15	Drug-free	Automatic speech SRRS DST/REM	Speech pause time Phonation time Cortisol levels REM latency
Nilsonne 1987 [30]	MEL = 8 (RDC) non-MEL = 8	Antidepressant Neuroleptics Lithium	Automatic speech CPRS	Speech pause time Phonation time FO
Nilsonne 1988 [31]	MEL = 21 (DSM III) non-MEL = 7	Antidepressant Neuroleptics	Automatic speech Free speech	Speech pause time Phonation time FO 5P
Kuny St. and Stassen 1993 [32]	MDD = 30 (ICD) Controls = 30	Antidepressant	Automatic speech HDRS	Speak flow Prosody
Flint et al. 1993 [33]	MDD = 30 (DSM III-R) +aged > 60 Controls = 30 Parkinsonian = 30	Antidepressant	Automatic speech HDRS	F2 Spirantization Voice onset time
Alpert et al. 2001 [34]	MDD = 12 (DSM III-R) +aged over 60 +HDRS ≥ 20	Sertraline Nortriptyline	Automatic speech Free speech HDRS	Fluency and prosody at day 0 and week 12
Cannizzaro et al. 2004 [35]	Depressed = 7 (NS) + HDRS ≥ 17	NS	Free speech HDRS	Speech pause time Phonation time FO
Hergueta et al. 1996 [36]	MDD = 40 (DSM IV) Controls = 40	Tricyclics IMAO SRRI	Gait analysis	Spatial and temporal parameters of gait
Lemke et al. 2000 [37]	MDD = 12 (DSM IV) Controls = 16	Amitriptyline Paroxetine Doxepin	Gait analysis	Stride length Gait velocity Double limb support
Hausdorff et al. 2004 [38]	MDD = 32 (DSM IV) Controls = 18	NS	Gait analysis	Stride time variability Swing time variability
Lecrubier 2006 [39]	Depressed = 26 (NS) Controls = 18	Antidepressant	Gait Analysis before and after treatment	Speed of propulsion of heel Stride length
Royant-Parola et al. 1986 [26]	UP = 12 (DSM III)	Tricyclics Mianserin Benzodiazepine	ActimetrySRRS	Level of activity Index of immobility
Dantchev et al. 1992 [25]	MDD = 13 (DSM III-R)	Trimipramine	ActimetrySRRS MADRS	Level of activity Index of immobility
Raoux 1994 [24]	MDD = 26 (DSM III-R) +MADRS > 25	Tricyclics	ActimetrySRRS	Level of activity Index of immobility
Volkers et al. 2003 [40]	MDD = 67 UP (DSM IV) Controls = 67	Drug-free	ActimetrySADS	Level of activity Fragmentation index
Iverson 2004 [41]	MDD = 48 (DSM IV) Controls = 25	NS	Actimetry	Level of activity
Lemke et al. 1997 [13]	MEL = 16 (DSM IV)	Antidepressant Benzodiazepine	ActimetryMAACL	Level of activity
Lemke and Schleidt 1999 [42]	MDD = 12 (DSM IV) Controls = 30	Amitriptyline	Video analysis of limb movements	Unit of action
Aybek et al. 2008 [43]	MDD = 4 (DSM IV) Controls = 7	NS	Movements of the limbs	Velocity and amplitude of the movements
van Hoof et al. 1993 [44]	MDD = 20 (DSM III-R) Controls = 20	Clomipramine Amitriptyline Neuroleptics	Drawing tasks SRRS	Movement time Reaction time Reinspection time
Sabbe et al. 1996 [45]	MDD = 22 (DSM III-R) Controls = 22	Fluoxetine Benzodiazepine Neuroleptic	Drawing tasks SRRS	Movement time Reaction time
Sabbe et al. 1999 [46]	MDD = 30 (DSM III-R) Controls = 30	Fluoxetine Benzodiazepine Neuroleptic	Drawing tasks	Movement time Velocity
Pier et al. 2004 [47]	MEL = 20 (DSM IV) non-MEL = 18 Controls = 38	Hypnotic	Drawing tasks SRRS	Movement time Reaction time Reinspection time
Pier et al. 2004b [48]	MDD = 12 (DSM IV) +age > 65 Controls = 12	Antidepressant Neuroleptic Mood stabilizer	Drawing tasks SRRS	Movement time Reaction time Reinspection time
Mergl et al. 2004 [49]	MDD = 37 (ICD 10)	Antidepressant Mood stabilizer	Drawing tasks Writing tasks	Kinematic parameters
Hoffstaedter et al. 2012 [50]	MDD = 20 (ICD 10) Controls = 20	Antidepressant Mood stabilizer Antipsychotic	Motor tasks Combined motor and cognitive measures	Reaction time Movement time Error rates
Schwartz et al. 1976 [51]	Depressed = 12 (NS) Controls = 12	None	Facial EMG during the generation of affective imagery	EMG patterns
Sweeney et al. 1998 [52]	MDD = 29 (DSM III-R)	None	Eye tracking	Latency of eye movements
Mahlberg et al. 2001 [53]	Depressed = 32 (NS) Controls = 42	NS	Eye tracking	Pro-saccades Predictive saccades
Winograd-Gurvich et al. 2006 [54]	MEL = 10 (DSM IV) non-MEL = 9	NS	Eye tracking	Latency of eye movements
Blackburn 1975 [55]	Depressive UP and BP: 106 (NS)	NS	Nufferno speed test Gibson spiral maze	Reaction time
Cornell et al. 1984 [56]	MEL = 14 (DSM III) non-MEL = 14 (DSM III) Controls = 14	None	Reaction choice test	“Motor” RT “Cognitive” RT
Smith et al. 1994 [57]	MDD = 36 (DSM III) Controls = 36	Antidepressant	Signal detection time SRRS MADRS	False alarms Omissions
Moffot et al. 1994 [12]	MEL = 20 (DSM III-R) Controls = 20	Antidepressant Mood stabilizer	Tests at 8 PM and AM DSST CANTAB Strength	Reaction time Movement time
Smith et al. 1995 [58]	MEL = 32 (DSM III-R) +MADRS > 22 Controls = 32	Antidepressant Benzodiazepine	Modified version of the Posner test SRRS	Reaction time
Brebion et al. 1995 [59]	MDD = 29 (DSM III-R) Controls = 26	Antidepressant Anxiolytic	Reaction time task SRRS MADRS	Reaction time
Brébion et al. 1997 [60]	MDD = 26 (DSM III-R) +MADRS > 20 Controls = 26	Antidepressant Benzodiazepine	Recognition memory task SRRS	Index of response bias Index of discrimination
Lemelin et al. 1996 [61]	MDD = 30 (DSM IV) Controls = 30	None	Stroop test SRRS	Reaction time Interference score
Lemelin and Baruch 1998 [62]	MDD = 30 (DSM IV) Controls = 34	None	Stroop test SRRS	Reaction time Interference score
Bonin-Guillaume et al. 2008 [63]	MDD = 16 (DSM IV) +GDS > 11 +age > 65 Controls = 16	NS	Reaction time task SRRS	Reaction time
Chen et al. 2013 [64]	MDD = 33 (DSM IV) Controls = 30	Antidepressant	Ego-rotation and object-rotation tasks	Reaction time Error rates
Caligiuri and Ellwanger 2000 [65]	MDD = 36 (DSM IV) Controls = 22	Antidepressant Neuroleptic Benzodiazepine	Wrist movements	Reaction Time Movement time
van Hoof et al. 1998 [66]	MDD = 20 (DSM III-R) Schizophrenic = 20	Antidepressant Neuroleptic Benzodiazepine	DSST SRRS	Observation time Writing time
Rogers et al. 1987 [67]	MDD = 30 (RDC) Parkinson = 20 Controls 30	Antidepressant Benzodiazepine Mood stabilizer Neuroleptic	DSST WBS	Reaction time Movement time
Rogers et al. 2000 [68]	MEL = 12 (DSM IV) non-MEL = 12 Controls = 24	Antidepressant Benzodiazepine Mood stabilizer	Reaction time task CORE	Reaction time
El Massioui et al. 1996 [69]	MDD = 8 (DSM III-R) +score SRRS > 27 Controls = 9	None	Event-related potential	Reaction time Amplitude and latency of P3, N1, and N2
Bange and Bathien 1998 [70]	MDD = 23 (DSM III-R) Controls = 20	Antidepressant Mood stabilizer	Event-related potential	Reaction tme Amplitude and latency of P3, N1, and N2
Schrijvers et al. 2009 [71]	MDD = 26 (DSM IV) Controls = 26	NS	EEG Drawing tasks	Error Negativity (Ne) Reaction time

BP: bipolar; UP: unipolar; CPRS: Comprehensive Psychopathological Rating Scale; CANTAB: computerised psychometric testing battery; DSM: Diagnostic and Statistic Manual of Mental Disorders; DST: suppression dexamethasone Test; DSST: digit symbol substitution test; EEG: electroencephalography; EMG: Electromyography; F0: Fundamental Frequency; HDRS: Hamilton Depression Rating Scale; ICD: International Statistical Classification of Diseases and Related Health Problems; MAACL: multiple affective adjective checklist; IMAO: monoamine oxydase inhibitor; GDS: Geriatric Depression Scale; MADRS: Montgomery Asberg Depression Rating Scale; MDD: major depressive disorder; MEL: melancholic; non-MEL: nonMelancholic; MT: movement time; NS: not specified; PMR: psychomotor retardation; PT: phonation time; RDC: Research Diagnosis Criteria; REM: rapid eye Movements; RT: reaction time; SADS: Schedule for Affective Disorders and Schizophrenia; SPT: speech pause time; SRRI: selective serotonin reuptake inhibitor; SRRS: Salpetrière Retardation Rating Scale; WBS: Webster Rating Scale.

**Table 2:** Studies exploring the physiopathology of psychomotor retardation.

Authors	Sample (criteria)	Treatments	Methods	Results
Bench et al. 1993 [72]	Depressed = 40 (RDC) Controls = 30	Antidepressant Neuroleptic Mood stabilizer	SPECT Item “retardation” SADS	Negative correlation between PMR and CBF in the LDPFC and angular gyrus
Hickie et al. 1995 [73]	MDD = 39 (RDC) Controls = 19	Antidepressant ECT Mood stabilizer	MRI Neuropsychological assessment (TMT, DSST)	Association PMR/white matter hyperintensities
Hickie et al. 1999 [74]	MDD = 25 (RDC)	NS	SPECT Reaction time	Negative correlation between reaction time and neostriatal blood flow
Videbech et al. 2002 [75]	MDD = 42 (DSM IV) Controls = 15	Antidepressant Neuroleptic Mood stabilizer	PET MRI SRRS	Negative correlation between SRRS and CBF in dorsolateral and supraorbital prefrontal cortices
Naismith et al. 2002 [76]	MDD = 46 (DSM IV) Controls = 20	Antidepressant	SPECT Stroop test	Negative correlation between PMR and blood flow in the LDPFC and angular gyrus
Walther et al. 2012 [77]	MDD = 20 (DSM IV) Controls = 19	Antidepressant Mood stabilizer Benzodiazepine Hypnotic	MRI Actigraphy	Positive association between activity level and CBF in the right orbitofrontal cortex and inverse association in the left supplemental motor area
Walther et al. 2012 [78]	MDD = 21 (DSM IV) Controls = 21	Antidepressant Mood stabilizer Benzodiazepine Hypnotic	Diffusion tensor imaging Actigraphy	Negative association between activity level and fractional anisotropy underneath the left primary motor cortex
Bracht et al. 2012 [79]	MDD = 21 (DSM IV) Controls = 21	Antidepressant Mood stabilizer Benzodiazepine Hypnotic	Diffusion tensor imaging Actigraphy	Alteration of white matter organisation of rostral anterior cingulate cortex-presupplementary motor area and dLPFC-presupplementary motor area pathways
Shah et al. 1997 [80]	MDD = 15 (DSM IV) Controls = 15	Antidepressant Benzodiazepine Mood stabilizer	IBZM-SPECT CANTAB HDRS	Negative correlation between IBZM binding and psychomotor speed, but not with the HDRS retardation item
Austin et al. 2000 [81]	MEL = 7 (DSM IV) Controls = 30	None	Single administration of the dopamine agonist apomorphine: motor and neuropsychological tests before and after injection	No improvement of motor and cognitive performance after apomorphine injection
Martinot et al. 2001 [82]	MDD = 12 (DSM IV) Controls = 7	SRRI	PET MRI	Retarded patients: reduction of fluorodopa uptake in the left caudate
Meyer et al. 2006 [83]	MDD = 21 (DSM IV)	None	PET Neuropsychological assessment	Correlation between putamen D_2_ binding potential and motor performances
Bajbouj et al. 2006 [84]	MDD = 20 (DSM IV) Controls = 20	None	TMS CORE	Reduced GABAergic tone in MDD
Mitchell et al. 1996 [85]	MEL = 20 (DSM IV/CORE) Controls = 20	Antidepressant Neuroleptic	Dexamethasone suppression test HDRS CORE	Negative correlation between CORE score and cortisol level
van Londen et al. 1997 [86]	MDD = 48 (DSM III-R) Controls = 30	Benzodiazepine	AVP concentrations Actimetry MADRS	Correlation between AVP concentrations and motor activity during wakefulness
van Londen et al. 1998 [87]	MDD = 52 (DSM III-R) Controls = 48	Benzodiazepine	AVP concentrations SRRS MADRS	Plasma AVP concentrations: severe retarded MDD > mild/no retarded MDD

AVP: arginine vasopressin; CANTAB: computerised psychometric testing battery; CBF: cerebral blood flow; DSM: Diagnostic and Statistic manual of Mental Disorders; DSST: digit symbol substitution test; ECT: electroconvulsivetherapy; IBZM-SPECT: iodo-methoxybenzamide-single photon emission tomography; HDRS: Hamilton Depression Rating Scale; LDLPFC: Left Dorsolateral Prefrontal Cortex; MEL: melancholic; MDD: major Depressive Disorder; MRI: magnetic resonance imaging; PET: positron emission Tomography; PMR: psychomotor retardation; RDC: Research Diagnosis Criteria; SADS: Schedule for Schizophrenia and Affective Disorder; SPECT: single photon emission computed tomography; SRRI: selective serotonin reuptake inhibitor; SRRS: Salpetrière Retardation Rating Scale; TMS: transcranial magnetic stimulation; TMT: trail making test.

**Table 3:** Studies exploring the predictive capacity of psychomotor retardation treatment response.

Authors	Design	Sample (criteria)	Intervention	Treatments associated	Criteria	Results
Del Zompo et al. 1990 [88]	Comparative, randomized trial	MDD = 60 (DSM-III) +HDRS > 16	(i) Minaprine: 6 weeks, 30 patients (ii) Amitriptyline: 6 weeks, 30 patients	Lorazepam	Item “retardation” HDRS	Minaprine: reduction of score on the item “retardation”
Rampello et al. 1991 [89]	Double blind, randomized, against placebo trial	UP = 40 (DSM III-R) +clinical retardation	(i) Amineptine: 4 weeks, 10 patients (ii) Minaprine: 4 weeks, 10 patients (iii) Placebo: 4 weeks, 10 patients (iv) Clomipramine: 4 weeks, 10 patients	None	HDRS SRRS	Minaprine and amineptine: reduction of score on SRRS
Burns 1995 [90]	Double blind, comparative trial	MDD = 183 (DSM III-R)	(i) Lofepramine: 6 weeks, 93 patients (ii) Fluoxetine: 6 weeks, 90 patients	Benzodiazepine	Item “retardation” HDRS	PMR predict lower response to lofepramine
Entsuah et al. 1995 [91]	Meta-analysis	MDD = 1222 (DSM III-R)	(i) Venlafaxine: 6 weeks (ii) Imipramine: 6 weeks (iii) Placebo	NS	Item “retardation” HDRS	Retarded depression: higher response rate with venlafaxine
Sabbe et al. 1996 [92]	Comparative trial	MDD = 22 (DSM III-R) +HDRS > 18 Controls = 22	(i) Fluoxetine: 6 weeks (ii) Drawing tasks	Anxiolytic Neuroleptic	HDRS at day 0 and week 6 SRRS RT and MT	Partial improvement
Flament et al. 1999 [93]	Comparative, multicenter, randomized, double blind trial	MDD = 286 (DSM III-R)	(i) Wash out: 1 week (ii) Sertraline: 6 weeks (iii) Fluoxetine: 6 weeks	Hypnotic Temazepam	Item “retardation” HDRS	Sertraline > fluoxetine in melancholic depression with PMR
Bondareff et al. 2000 [94]	Comparative, randomized, double blind trial	MDD = 144 (DSM III-R) +age > 60 +MMSE > 24	(i) Wash out: 1 week (ii) Sertraline: 12 weeks (iii) Nortriptyline: 12 weeks	None	HDRS Neuropsychological assessments	Baseline information processing Resp = non-Resp Baseline executive functioning Resp > non-Resp
Navarro et al. 2001 [95]	Simple blind, randomized trial	MDD = 58 (DSM IV) +age > 60 +MMSE > 25	(i) Wash out: 2 weeks (ii) Nortriptyline: 12 weeks (iii) Citalopram: 12 weeks	Haloperidol	Item “retardation” HDRS	Severe retardation: response rate nortriptyline (82%) > citalopram (11%) Mild retardation: equal response rates (95 and 100%)
Ferguson et al. 2003 [96]	Comparative, multicenter, randomized, double blind trial	MDD = 350 (DSM III-R) +HDRS > 20	(i) Wash out: 4 to 28 days (ii) Reboxetine: 4–8 weeks, 350 patients (iii) Placebo: 353 patients	None	Item “retardation” HDRS	Reboxetine: early psychomotor improvement
Volkers et al. 2002 [97]	Comparative, randomized, double blind trial	MDD = 52 (DSM IV)	(i) Wash out: 7 days (ii) Imipramine: 4 weeks, 25 patients (iii) Fluvoxamine: 4 weeks, 27 patients	None	Actimetry SRRS	Imipramine: increase in daytime motor activity Fluvoxamine: no modifications in motor activity
Caligiuri et al. 2003 [98]	Double blind, randomized trial	MDD = 28 (DSM IV)	(i) Phenelzine: 8 weeks, 12 patients (ii) Sertraline: 8 weeks, 9 patients (iii) Bupropion: 8 weeks, 7 patients	None	Wrist rotation Item “retardation” HDRS	Baseline motor impairment: Resp < non-Resp
Sechter et al. 2004 [99]	Double blind, randomized, multicenter trial	MDD = 302 (DSM IV) +MADRS > 20	(i) Milnacipran: 6 weeks 148 patients (ii) Paroxetine: 6 weeks, 151 patients	None	Item “retardation” HDRS	Baseline PMR predict good response to milnacipran
Taylor et al. 2006 [100]	Open study	MDD = 47 (DSM IV)	(i) Wash out: 1 week (ii) Fluoxetine: 12 weeks	NS	COWAT FAS Stroop test	Baseline Resp COWAT FAS performance: non-Resp
Mallinckrodt et al. 2007 [101]	Meta-analysis	MDD = 2463 (DSM IV)	(i) Duloxetine: 8 weeks (ii) Escitalopram: 8 weeks (iii) Paroxetine: 8 weeks	NS	Item “retardation” HDRS	Greater reduction of PMR in duloxetine group
Herrera-Guzmán et al. 2008 [102]	Open study	MDD = 26 (DSM IV) +age > 60	Bupropion: 8 weeks	None	HDRS CANTAB	Psychomotor speed predicts response to bupropion
Singh et al. 2013 [103]	Double blind, randomized, multicenter trial	MDD = 113 (DSM IV)	(i) Venlafaxine: 8 weeks (ii) Escitalopram: 8 weeks	None	Item “retardation” HDRS CORE	Greater reduction of PMR in venlafaxine group PMR does not predict response to carbamazepine
Jouvent et al. 1998 [104]	Double blind, randomized, multicenter trial	MDD = 124 (DSM IV) +MADRS > 25 +SRRS > 20	(i) Moclobemide: 4 weeks, 60 patients (ii) Clomipramine: 4 weeks, 59 patients	None	SRRS at days 7, 10, and 14	Moclobemide: reduction of SRRS score at day 7
Joffe et al. 1987 [105]	Open study	Depressed = 19 (RDC)	Carbamazepine	None	Actimetry	PMR does not predict response to carbamazepine
Álvarez et al. 1997 [106]	Open study	MDD = 105 (DSM III-R)	Lithium	Imipramine or equivalent	Item “retardation” NDI	PMR does not predict response
Hantouche et al. 2005 [107]	Retrospective study	MDD = 59 (DSM IV)	(i) Lithium (ii) Valpromide (iii) Carbamazepine	NS	Item “retardation” HDRS	Lower response rate to mood stabilizer in motor-retarded patients
Strian et al. 1979 [108]	Longitudinal study	MDD = 36 (ICD)	ECT	NS	Item “retardation” and “agitation” HDRS	Early improvement in “agitated” group Mood fluctuations in “retarded” group
Hickie et al. 1990 [109]	Open study	MDD = 36	ECT: unilateral	Antidepressant Antipsychotic	CORE	CORE predict response to ECT
Buchan et al. 1992 [110]	Comparative study	MDD = 165 (NS)	(i) Real ECT: 2 sessions per week, 4 weeks (ii) Sham ECT: 2 sessions per week, 4 weeks	Anxiolytic	PSE	Improvement in patients with PMR
Sobin et al. 1996 [111]	Randomized, double blind study	MDD = 148 (RDC)	(i) Real ECT: 3 sessions per week (ii) Sham ECT: 3 sessions per week	Lorazepam	HDRS day 0 and every week	Response rate: “retarded” = “non retarded”
Hickie et al. 1996 [112]	Open study	MDD = 81 (NS)	ECT: 10 sessions	Antidepressant Antipsychotic Benzodiazepine Mood stabilizer	CORE	CORE predicts response to ECT
Höppner et al. 2003 [113]	Randomized, against placebo trial	MDD = 30 (DSM IV) +MADRS > 18 Controls: 30	(i) TMS: high frequency over the right DLPFC, 10 sessions (ii) TMS: low frequency over the left DLPFC, 10 sessions (iii) Sham TMS	Antidepressant	MARS	Early improvement of psychomotor performance in the “high frequency” group
Höppner et al. 2010 [114]	Comparative, randomized, double blind trial	MDD = 30 (DSM IV) +MADRS > 18 Controls: 30	(i) TMS: low frequency over the left DLPFC, 10 sessions (ii) Sham TMS	Venlafaxine Mirtazapine Lorazepam	MARS	No effect of TMS on PMR
Baeken et al. 2010 [115]	Open study	UP = 20 (DSM IV) +resistance criteria	TMS: over the left DLPFC, 10 sessions	Benzodiazepine Neuroleptic	SRRS	Improvement of psychomotor performance
Ullrich et al. 2012 [116]	Double blind, placebo controlled randomized	MDD = 43 (DSM IV)	TMS: over the left DLPFC, 15 sessions, ultrahigh frequency	Lithium Venlafaxine Mirtazapine Antipsychotic Benzodiazepine	Item “retardation” HDRS	Improvement of psychomotor performance
Loo et al. 2010 [117]	Double blind, placebo controlled randomized	MDD = 40 (DSM IV)	tDCS: 10 sessions of anodal tDCS over the left DLPFC, at 1 mA	None	CORE MADRS	No significant difference in depression scores after real compared with sham tDCS No improvement in CORE score
Loo et al. 2012 [118]	Double blind, placebo controlled randomized	MDD = 64 (DSM IV)	tDCS: 15 sessions of anodal tDCS over the left DLPFC, at 2 mA	None	CORE MADRS	Significant difference in depression scores after real compared with sham tDCS No improvement in CORE score

CANTAB: computerised psychometric testing battery; DLPFC: dorsolateral prefrontal cortex; DSM: Diagnostic and Statistical manual of mental disorders; ECT: electroconvulsivetherapy; HDRS: Hamilton Depression Rating Scale; MADRS: Montgomery Asberg Depression Rating Scale; MARS: Motor Agitation and Retardation Scale; MDD: major depressive disorder; MMSE: Mini Mental State Examination; NDI: Newcastle Index of Depression; NS: not specified; PMR: psychomotor retardation; PSE: Present State Examination; Resp: Responder; RDC: Research Diagnosis Criteria; SRRI: selective serotonin reuptake inhibitor; SRRS: Salpetrière Retardation Rating Scale; UP: unipolar
